# The performance evaluation of management mode of small water resources projects

**DOI:** 10.1371/journal.pone.0282357

**Published:** 2023-04-06

**Authors:** Shengteng Qu, Huan Chen, Zhuge Shen, Haoxiang Ma

**Affiliations:** 1 Business School of Hohai University, Nanjing, China; 2 College of Civil Engineering, Hefei University of Technology, Hefei, China; 3 Huai’an Financial Center Investment and Construction Co., Ltd, Huai’an, China; Xiangtan University, CHINA

## Abstract

Due to a series of societal factors, management of small rural water resources projects in China experience management problems. Based on the management mode of small water resources projects in three representative regions of Guangdong Province, the improved TOPSIS model is applied to evaluate the performance of management mode of small water resources projects through the combination with entropy weight method. Compared with the traditional TOPSIS model concerning the evaluation object of this paper, the evaluation value formulas of optimal and worst solutions of TOPSIS method are improved. The evaluation index system takes into account the coverage, hierarchy and systematization of indicators, and maintains a management mode with high environmental adaptability, so that the continuous operation of management mode can guaranteed. The results show that the management mode of water user association is most suitable for the development of small water resources projects in Guangdong Province.

## Introduction

During the 1950s and 1960s, the Chinese government built small-scale water resources projects in a large quantity through investment and farmers’ devotion of labor, and these measures were of great significance for irrigation and drainage. However, with the passage of time, most of the projects have fallen into disrepair, thus required to be rebuilt urgently. In addition, some reservoirs have been unable to work normally, and turned out to be safety-risk projects with some constant problems, especially the problems of embankment collapse, river siltation, channel leakage, lacking the function of water storage and drinking water source pollution for small-scale reservoirs. Most of the work related to building, operating and maintaining water projects is completed by farmers, but there is no clear organizational structure for them to work as a group. As a result, it leads to unclear property rights, a lack of participation in collective efforts, and farmers’ inability to understand their identity as water managers. Small-scale village-level water resources projects are almost unsupervised, which has become a common problem of rural development in China in recent years. To solve the key problems related to management in small-scale water resources projects at the village level, China has gradually conducted reform for the management system of some small-scale water resources projects abiding by the principle that “anyone who benefits takes the responsibility and who invests takes possession of it”. It is carried out based on observing the general law of the socialist market economy and the law of water resources development in the rural area [1, 2]. It is mainly manifested in the construction of cooperative organizations for water use, the clarification of ownership, and the introduction to various methods of management, to improve farmers’ participation in the management of water resources projects [2–4]. Guided by this policy, China has gradually established typical modes applied in the management of small-scale water resources projects, such as joint-stock cooperative system, water users associations, and governmental domination.

The joint-stock system adopted by small-scale water resources projects is established to satisfy the demands of rural areas in the construction of a new diversified system for water resources investment. It is conducive to construction and management, as well as the increase in social benefits [5–7]. The model of Water Users Association is a cooperative model in which water users of farmers voluntarily form a cooperative relationship with each other. As a result, it stimulates farmers’ passion for the management of water resources projects, in addition to solving the problem related to the lack of project management to a certain extent, due to farmers’ high-degree of participation [7–10]. The government-dominated management mode of small-scale water resources projects refers to the government’s use of financial subsidies to effectively manage small-scale water resources projects. It is surely conducive to ensuring the effective management of small-scale water resources projects at the village level, thus promoting agricultural production and income [11–13]. Targeting at the characteristics shown by different rural areas, the management mode can be selected, so that the small-scale water resources projects at village level can realize their long-term benefits. The research conducted by Zhang et al. [14] shows that the factors, including farmers’ cultural level, project familiarity, information sharing and so on in a region, have a positive influence on the individual farmers’ willingness to participate in project management. To judge which management mode is the most appropriate for small-scale water resources projects in an area, the evaluation over management performance of several modes is in high demand [15].

Economic theory reflects that the more efficient the management mode is, the faster the total output can grow. TOPSIS (Technique for Order Preference by Similarity to Ideal Solution) model proposed by Hwang and Yoon in 1981 is a decision-making technique which belongs to the analysis on the multi-objective decision making with finite scheme in systematical engineering. It is also a method employed in distance comprehensive evaluation. TOPSIS has produced many engineering application cases in recent years. Yang [16] used TOPSIS to establish a comprehensive evaluation model of six location indicators, such as river width and average annual precipitation, to scientifically locate the dam and improve the quality of hydraulic engineering. Lu et al. [17] solved design and planning evaluation problems by using fuzzy TOPSIS, which includes not only quantitative calculation but also qualitative analysis, and can be an effective method for construction method decision-making.

Specifically, the research in this paper has the following theoretical and practical contributions:1. To make comparison between the advantages and disadvantages of the management modes adopted by several small-scale water resources projects of the typical type and obtain the detailed results of performance evaluation shown by the mode in specific areas, this paper selects three representative objects of evaluation in Jiaoling, Gaozhou and Nanhai from Guangdong Province. 2. The improved TOPSIS model is used to evaluate the performance of the management mode of small-scale water resources projects. Consequently, it will be beneficial to not only knowing the best management model in Guangdong Province but also applying the model to similar projects around the world. 3. The establishment of a performance evaluation system for small-scale water resources projects and the discussion of different management modes can provide theoretical and practical guidance for performance evaluation of similar small water resources projects around the world.

## Discussion

With regard to small water resources, the reservoir capacity is small, the installed capacity of hydropower station is small, and the irrigation area is small. In comparison with large-scale water resources projects, small-scale water resources projects have low complexity due to its small scale. If a standardized management system is established, its management efficiency will be greatly improved. Therefore, the analysis on specific situation of the three typical management modes is carried out, and specific management problems in each situation are obtained, which provides theoretical support for the establishment of the index system in the following part of this article.

### Joint-stock management mode

In the mode of shareholding management, the rights of ownership, use, income, and disposal of small-scale water resources projects are relatively concentrated, influenced by the investment structure. Accordingly, two cooperative groups consisting of “core members” and “peripheral members” are formed within the cooperative. With heavy investment, “core members” hold a large proportion of the shares, thus occupying a dominant position in the cooperatives, while “peripheral members” rely on core members owing to their lack of the right of speech. Therefore, in the benefit game between “core members” and “peripheral members”, it is inevitable that the system design tends to pay more attention to the core members. Hence, it is rather difficult for peripheral members to protect their own interests by uniting as a whole. Some government-initiated cooperative supervisory boards and member meetings are not held timely, so that ordinary members fail to understand the process of decision making, and therefore they lack the channels to express the request of their own interests effectively. Inadequate supervision weakens the binding effect of the contract on the management layer, and farmers would face difficulty in truly understanding the operation status of the cooperative due to the lack of market-related information that can transmit the internal management level of the cooperative. Therefore, it is likely that only a small number of core members in the cooperative can control the decision-making process of business. Then, the decisions unfavorable to farmer households or ordinary members and the situation where capital exploits labor and large households exploit small households will arise. Hence, in the performance evaluation of the management mode, the high quality of the supervision mechanism is an important reference indicator.

### Water users association management mode

Under the management mode of Water Users Association, the right of management is separated from the right to maintain. The right to use a small-scale water resources project belongs to the association responsible for the project. It takes more time and energy for the government to manage, plan and formulate policies from a macro perspective. Therefore, WSA and the government are in high demand of establishing a new water commodity trade relationship and partnership in which mutual support and equal cooperation are available. Based on the above analysis, the performance evaluation of this management mode is composed of two parts. On the other hand, the bureau responsible for the management of small-scale water resources projects assumes the responsibility of water supply, and its implementation efficiency has close relationship with the vital interests of farmers. Because it is an important factor affecting farmers’ behavior, it should also be included in the evaluation system.

### Government-dominated management mode

In this mode, property rights are concentrated. The government makes appropriate investment in the construction of related small-scale water resources projects in the ways of investment and subsidies etc., and it is owned by the government and the collective. The agencies in charge of the management of small-scale water resources projects are responsible for the daily operation projects, as well as the rights of management and operation. The work related to maintenance and management under this mode is also conducted by the government collectively. It is easy to see that the government, under this mode, serves as a provider of public services and it plays an important role in the construction, operation and maintenance of small-scale water resources projects. Moreover, the goals pursued by the government include not only economic but also social and ecological benefits.

A scenario analysis on three representative small-scale water resources projects is conducted by us. The management problems of small-scale water resources projects are analyzed according to three specific scenarios. In the first shareholding management mode of small-scale water resources projects, the ownership, use, usufruct and disposal rights of small-scale water resources projects are relatively concentrated, so it is necessary to consider whether the supervision mechanism is perfect when designing indicators. In the second management mode—water users association, the right of management is separated from the right of maintenance, so the implementation efficiency of water supply responsibility is included in the index design. In the third government-led management mode of small-scale water resources projects, property rights are relatively concentrated, so economic and social benefits are considered in the design of indicators.

### Selection of evaluation indicators

On the one hand, reforming the management mode adopted by small-scale water resources projects has improved the performance of water resource management in rural areas. On the other hand, reform has increased utilization efficiency of irrigation water, improved the success rate of small-scale agricultural water projects, and alleviated the serious shortage of related funding. The reform of the management mode plays a non-negligible role in small-scale water resources projects, which is of vital importance in the management of China’s farmland. Therefore, to evaluate the performance of farmers’ water cooperatives, it is necessary to establish a comprehensive, explicit, and scientific system of indicators. Moreover, the establishment of a performance evaluation index system is the core part of the performance evaluation and a key step which ensures the credibility of the results obtained from the evaluation.

The management of small-scale water resources projects covers many factors including engineering, technology, management, economy, and environment, and therefore the evaluation index system should consider many factors so that the evaluation indicators can fully reflect the status and effect shown by the management of small-scale water resources projects. Apart from taking into account the coverage, the evaluation index system should consider the hierarchical and systematic nature of the indicators. Additionally, the setting of index should focus on both its sustainability and the standardized operation of the management mode. Only when the management mode is sustainable and highly adaptable to the environment, can it be operated continuously. In the process, the coverage, hierarchy and systematization of indicators are comprehensively considered, in addition to the principles of being scientific, comprehensiveness, non-intersection and easy access, followed by a large number of literature review and expert interviews. The system is divided into organizational management, engineering management, water management, economic management and sustainability management based on the index division of some references and the domain knowledge of experts.

According to corresponding references and experts’ theories [18–22], a performance evaluation system for the management mode of small-scale water resources projects has been formed. Covering three levels, the index system is composed of one target layer, the performance evaluation for the management mode of small-scale water resources projects, 5 system layers—organizational management, project management, water management, economic management, and sustainable management, and the index layer including 27 indicators. In terms of indicator types, they are divided into qualitative and ration types. The organizational management divided in this study is biased towards the content of static organizational management, the design of organizational structure and mechanism, while the project management system pays more attention to the problems in the process and dynamic indicators in the management of small-scale water resources projects. Therefore, in the scenario set by this study, the relationship between organizational management and engineering management system is a combination of dynamic and static. Qualitative indicators refer to assessment indicators that cannot be directly quantified, while ration indicators can be quantitatively defined and measured with accuracy. Positive indicator means “bigger is better”, while negative indicator means “smaller is better”. Please refer to Table 1 for details.

**Table 1:** Performance evaluation system of management mode of small-scale water resources projects.

Overall Layer	System Layer	Indicator Layer	Units	Indicator Type	Direction
Performance evaluation of small-scale water resources project management mode (*A*)	Organization management (*B*_1_)	Institutional setting (*C*_11_)	-	Qualitative	positive
		Incentive Reward and Punishment Mechanism (*C*_12_)	-	Qualitative	positive
		Supervisory mechanism (*C*_13_)	-	Qualitative	positive
		Democratic Decision-Making Mechanism(*C*_14_)	-	Qualitative	positive
		Archives Management (*C*_15_)	-	Qualitative	positive
	Project Management (*B*_*2*_)	Engineering Property Rights Clarity (*C*_21_)	%	ration	positive
		Custody Responsibility Fulfillment Rate (*C*_22_)	%	ration	positive
		Water source engineering integrity (*C*_23_)	%	ration	positive
		Channel uptime (*C*_24_)	%	ration	positive
		Electromechanical equipment uptime (*C*_25_)	%	ration	positive
		Gutter integrity (*C*_26_)	%	ration	positive
	Water Management (*B*_*3*_)	Frequency of water conflicts (*C*_31_)	%	ration	negative
		Water cycle reduction rate (*C*_32_)	%	ration	positive
		Irrigation water use efficiency (*C*_33_)	%	ration	positive
		Standardize water registration rate (*C*_34_)	%	ration	positive
		Water Plan Completion Rate (*C*_35_)	%	ration	positive
	Economic Management (*B*_*4*_)	Water Pricing Rationality (*C*_41_)	-	Qualitative	positive
		Water charge rate (*C*_42_)	%	ration	positive
		Water price cost ratio (*C*_43_)	%	ration	negative
		Water charges use transparency (*C*_44_)	%	ration	positive
		Fixed asset depreciation rate (*C*_45_)	%	ration	positive
	Sustainable Management (*B*_*5*_)	Water Security Ratio (*C*_51_)	%	ration	positive
		Funding Guarantee Rate (*C*_52_)	%	ration	positive
		Economic self-reliance rate (*C*_53_)	%	ration	positive
		Farmer satisfaction rate (*C*_54_)	%	ration	positive
		Water quality compliance rate (*C*_55_)	%	ration	positive
		Fund surplus growth rate (*C*_56_)	%	ration	positive

### Weight calculation of evaluation index

Through analysis and comparison, it is obvious that the entropy weight method is an objective method of empowerment, which determines the weight of the indicator using the size of the amount of information offered by the entropy value of each indicator [23]. Using the indicators, the entropy weight method is able to be free from human interference in the weights of each evaluation index, thus ensuring more realistic evaluation results. Moreover, it overcomes the problem—human factors affect the empowerment process of indicators to a severe extent in the evaluation method at this stage. In addition, through the calculation of the entropy value of each indicator, the measurement over the size of the index information can be conducted to ensure that the index established can reflect most of the original information. Hence, this paper calculates the index weight applying the entropy weight method.

(1) Standardized calculation of data: the difference in the meanings and purposes of each evaluation index usually lead to different dimensions and order of magnitudes in each indicator. Therefore, it is necessary to conduct non-quantitative treatment of the indicators in each subsystem to eliminate the dimensional influence on the results of evaluation and reduce the interference of random factors. The standardized formula is as follows.

$$r_{ij}=\frac{x_{ij}-\min(x_{ij})}{\max(x_{ij})-\min(x_{ij})}$$ (1)

In the equation, *x*_*ij*_ refers to the *i*th indicator value of the *j*th management mode. Moreover, *i* = 1,2…*m*, *j* = 1,2…*n*, where *m* denotes the number of indicators evaluated; *n* represents the number of management modes to be evaluated; *r*_*ij*_ indicates a standardized indicator. According to the calculations mentioned above, a standardized matrix is listed as follows:

$$R=\left[\begin{array}{l} r_{11}\;r_{12}\;\cdots \;r_{1n}\\ r_{21}\;r_{22}\;\cdots \;r_{2n}\\ \vdots \;\vdots \;\vdots \;\vdots \\ r_{m1}\;r_{m1}\;\cdots \;r_{mn} \end{array}\right]$$ (2)

(2) Calculate the information entropy of the *i*th indicator: the formula used in the calculation of entropy value is as follows:

$$H_{i}=-\frac{1}{\ln\;n}\sum_{j=1}^{n}f_{ij}\;\ln\;f_{ij}$$ (3)

In the equation, $f_{ij}=\frac{r_{ij}}{\sum_{j=1}^{n}r_{ij}}$ refers to the characteristic weight of the indicator.

(3) Calculate the entropy weight of the *i*th indicator:

$$w_{i}=\frac{1-H_{i}}{m-\sum_{i=1}^{m}H_{i}}$$ (4)

### Evaluation model building

In recent years, the TOPSIS has been applied in a variety of fields including risk decision-making analysis, environmental quality monitoring and assessment, evaluation of land ecological security and benefit evaluation of land consolidation implementation, with favorable achievement realized [24–26]. The advantages of this model lie in the ability to make full use of the original data, small amount of data loss in the computational process, and the intuitive geometric significance without the interference from reference sequence selection. “Positive ideal solution” and “negative ideal solution” are two important concepts in the TOPSIS model. During the process, there is a construction of two-dimensional data space to represent the distance between the optimal solution and the worst solution. Moreover, a research on the optimal solution and the worst solution in each evaluation index is usually conducted. Besides, there is the comparison between the evaluation index with the optimal solution and the worst solution based on the above-mentioned process. If the solution is the closest to the optimal solution and it is farthest away from the worst solution at the same time, then this solution is an optimal solution in the schemes to be evaluated. Conversely, if the solution is the closest to the worst solution and it is the farthest from the optimal solution, the scheme is the worst among the schemes to be evaluated.

Entropy method is so objective that it can reduce the deviation resulting from subjective assignment. As a commonly used method of multi-objective decision analysis, TOPSIS is suitable for multiple schemes. Entropy weight TOPSIS is to calculate the objective weight of the index using entropy weight method, followed by the evaluation of each evaluation object using the method of TOPSIS. The improved method reduces the human error caused by subjective assignment and increases the objectivity of index weight. In this paper, the evaluation of the performance shown by the management mode of small-scale water resources projects is conducted using an improved TOPSIS model in combination with the entropy method. In comparison with the traditional TOPSIS model, an improved TOPSIS model mainly shows its improvement in the formula of evaluation value for the treatment of the evaluation object using the optimal solution and the worst solution. Detailed steps of the implementation steps are listed as follows:

(1) Standardization: referring to the formula of data standardization offered in the previous subsection, the index data of the evaluation object are standardized to reduce the interference from random factors.
(2) Determination of metric weights: weighted thinking facilitates the matrix *Y*, the weighted normalized evaluation, and it is to be constructed using *w*_*i*_, which can be obtained by multiplying matrix *R* in each row with its weight *w*_*i*_:

$$Y=\left[\begin{array}{c} y_{11}\;y_{12}\;\cdots \;y_{1j}\\ y_{21}\;y_{22}\;\cdots \;y_{2j}\\ \vdots \;\vdots \;\vdots \vdots \\ y_{i1}\;y_{i1}\;\cdots \;y_{ij} \end{array}\right]=\left[\begin{array}{c} r_{11}\;w_{1}\;r_{12}\;w_{1}\;\cdots \;y_{1n}\;w_{1}\\ r_{21}\;w_{2}\;r_{22}\;w_{2}\;\cdots \;y_{2j}\;w_{2}\\ \vdots \;\vdots \;\vdots \;\vdots \\ r_{m1}\;w_{m}\;r_{m2}\;w_{m}\;\cdots \;r_{mn}\;w_{m} \end{array}\right]$$ (5)

(3) Seeking for positive and negative ideal solutions: Let *Y*^*+*^ be the maximum value of the *i*th indicator in the *j* management mode, and it is the positive ideal solution, the most ideal solution as well. Let *Y*^*-*^ be the minimum value of the *i*th indicator in the *j*th management mode, and it is the negative ideal solution, the least ideal solution as well. The formulas used in calculation are as follows:

$$Y^{+}=\{\max\;y_{ij}|i=1,2\ldots m\}=\{y_{1}^{+},y_{2}^{+}\ldots y_{m}^{+}\}$$ (6)

$$Y^{-}=\{\min\;y_{ij}|i=1,2\ldots m\}=\{y_{1}^{-},y_{2}^{-}\ldots y_{m}^{-}\}$$ (7)

(4) Distance calculation: calculate the distance of the *i*th indicator from the positive and negative ideal points (*Dj+*, *Dj-*) in different management modes *j*, with the calculation formulas listed as follows:

$$D_{j}^{+}=\sqrt{\sum_{i=1}^{m}(y_{i}^{+}-y_{ij}{)}^{2}}$$ (8)

$$D_{j}^{-}=\sqrt{\sum_{i=1}^{m}(y_{i}^{-}-y_{ij}{)}^{2}}$$ (9)

In the equation, *y*_*i*_^*+*^ and *y*_*i*_^*-*^ refer to the most preferred and least preferred values for the *i*th indicator, respectively.

(5) Calculating *T*_*j*_, the degree of closeness shown by different models to the optimal solution: the closeness denotes the degree to which *T*_*j*_ approaches the optimal management mode in the *j*th management mode, with the value range of [0, 1]. The greater the value of *T*_*j*_ is, the closer it is to optimal. The size of the closeness is used to judge the management mode performance, and therefore the order of advantages and disadvantages is determined. The formula used in calculation is as follows:

$$T_{j}=\frac{D_{j}^{-}}{D_{j}^{+}+D_{j}^{-}}$$ (10)

In the equation, the greater the value of *T*_*j*_ is, the closer the *j*th index in the management mode of different small-scale water resources projects is to the optimal value, with the value range of *T*_*j*_ from 0 to 1. When the value of *T*_*j*_ is 1, it indicates an optimal jth indicator, and when the value of *T*_*j*_ is 0, it shows that the worst jth indicator. According to the research conducted by Sun et al. [19], Rouyendegh et al. [21] and others, *T*_*j*_ is divided into four levels for the qualitative evaluation of the management performance shown by small-scale water resources projects under different management modes, as shown in Table 2.

**Table 2:** Performance evaluation criteria for the management mode of small farmland water.

Closeness (*T*_*j*_)	Different closeness levels correspond to each other
[0,0.3]	poor
[0.3,0.6]	medium
[0.6,0.8]	good
[0.8,1]	excellent

## Case study

### Evaluate case selection

The performance evaluation of the management mode shown by small-scale water resources projects is required to be fully integrated with the actual situation of each place. On the basis of the research conducted previously, this paper selects three representative evaluation objects, including Jiaoling, Gaozhou and Nanhai in Guangdong Province, which are dominated by the joint-stock management mode, the management mode of the Water Users Association, and the government-led management mode, respectively. These three management modes are universally representative of the management modes currently applied in the major small-scale water resource projects in Guangdong Province. In the research, with the aim of enhancing the applicability of the index system to the performance evaluation of small-scale water resources projects under different modes, evaluation emphases of different dimensions from the three modes are selected to form the index system together. Therefore, the range of evaluation for this index system is wide, and horizontal comparison can be conducted for different small-scale water resources projects.

Covering all types of small-scale water resource projects, the study includes cooperatives, water users associations, management bureaus, and other responsible units. Then, water farmers, village committee members, and management bureau staff participating in the daily operation and management of small-scale water resources projects are interviewed with. Through a visit to the actual situation of the three typical areas, basic data on small-scale water resources projects were collected in a large amount. During the entire process of the survey, a total of 29 small forums were held for village cadres, peasant households and large-scale business households, with more than 900 people included. Besides, a total of 759 questionnaires were distributed, including 256, 243 and 260 pieces of questionnaires in the Jiaoling area, Gaozhou area, and Nanhai area respectively. There was a total recovery rate of 83%. After screening, 539 valid questionnaires were collected, and the ratio for the effectiveness of questionnaires was about 85%.

### Data processing

Having been preliminarily processed, the data collected by the survey is standardized according to the standardized formula to remove the impact of different dimensions. The results obtained from specific standardized processing are shown in Table 3 as follows.

**Table 3:** Basic data standardization on indicators of evaluation area.

Overall Layer	System Layer	Indicator Layer	Jiaoling	Gaozhou	Nanhai
Performance evaluation of small-scale water resources project management mode (A)	Organization management (B1)	Institutional setting (C11)	0.4078	0.5754	0.4561
		Incentive Reward and Punishment Mechanism (C12)	0.5141	0.6903	0.2674
		Supervisory mechanism (C13)	0.5331	0.6678	0.2949
		Democratic Decision-Making Mechanism (C14)	0.6541	0.7045	0.2501
		Archives Management (C15)	0.4026	0.3647	0.6107
	Project Management (B2)	Engineering Property Rights Clarity (C21)	0.5522	0.4362	0.4732
		Custody Responsibility Fulfillment Rate (C22)	0.3063	0.6585	0.4985
		Water source engineering integrity (C23)	0.0887	0.8366	0.7229
		Channel uptime (C24)	0.1329	0.8004	0.6849
		Electromechanical equipment uptime (C25)	0.0713	0.8507	0.6797
		Gutter integrity (C26)	0.0470	0.8414	0.8707
	Water Management (B3)	Frequency of water conflicts (C31)	0.1639	0.9108	0.5427
		Water cycle reduction rate (C32)	0.3437	0.8014	0.1316
		Irrigation water use efficiency (C33)	0.4469	0.6527	0.3133
		Standardize water registration rate (C34)	0.2179	0.6893	0.7308
		Water Plan Completion Rate (C35)	0.3323	0.5672	0.6372
	Economic Management (B4)	Water Pricing Rationality (C41)	0.6599	0.5167	0.3046
		Water charge rate (C42)	0.3430	0.2598	0.6965
		Water price cost ratio (C43)	0.6672	0.4429	0.3632
		Water charges use transparency (C44)	0.3557	0.6181	0.5103
		Fixed asset depreciation rate (C45)	0.0464	0.8712	0.2442
	Sustainable Management (B5)	Water Security Ratio (C51)	0.2850	0.6759	0.6367
		Funding Guarantee Rate (C52)	0.1574	0.4552	0.7803
		Economic self-reliance rate (C53)	0.1109	0.2714	0.8183
		Farmer satisfaction rate (C54)	0.3150	0.6973	0.2589
		Water quality compliance rate (C55)	0.4940	0.5522	0.4362
		Fund surplus growth rate (C56)	0.0818	0.8422	0.3594

*w*_*i*_, the entropy weight of each index, is calculated and multiplied by the standardized matrix to construct matrix *Y*, the weighted normalized evaluation, which is shown in Table 4.

**Table 4:** Weighted normalized evaluation matrix based on entropy weight method and TOPSIS.

Overall Layer	System Layer	Indicator Layer	Jiaoling	Gaozhou	Nanhai
Performance evaluation of small-scale water resources project management mode (A)	Organization management (B1)	Institutional setting (C11)	0.0013	0.0018	0.0014
		Incentive Reward and Punishment Mechanism (C12)	0.0101	0.0136	0.0053
		Supervisory mechanism (C13)	0.0080	0.0101	0.0044
		Democratic Decision-Making Mechanism (C14)	0.0159	0.0171	0.0061
		Archives Management (C15)	0.0032	0.0029	0.0048
	Project Management (B2)	Engineering Property Rights Clarity (C21)	0.0008	0.0006	0.0007
		Custody Responsibility Fulfillment Rate (C22)	0.0041	0.0088	0.0067
		Water source engineering integrity (C23)	0.0062	0.0586	0.0507
		Channel uptime (C24)	0.0072	0.0431	0.0369
		Electromechanical equipment uptime (C25)	0.0055	0.0658	0.0526
		Gutter integrity (C26)	0.0042	0.0753	0.0779
	Water Management (B3)	Frequency of water conflicts (C31)	0.0086	0.0479	0.0285
		Water cycle reduction rate (C32)	0.0224	0.0521	0.0086
		Irrigation water use efficiency (C33)	0.0058	0.0085	0.0041
		Standardize water registration rate (C34)	0.0069	0.0217	0.0230
		Water Plan Completion Rate (C35)	0.0034	0.0058	0.0065
	Economic Management (B4)	Water Pricing Rationality (C41)	0.0090	0.0070	0.0041
		Water charge rate (C42)	0.0093	0.0070	0.0188
		Water price cost ratio (C43)	0.0066	0.0044	0.0036
		Water charges use transparency (C44)	0.0026	0.0045	0.0037
		Fixed asset depreciation rate (C45)	0.0059	0.1104	0.0310
	Sustainable Management (B5)	Water Security Ratio (C51)	0.0051	0.0122	0.0115
		Funding Guarantee Rate (C52)	0.0076	0.0219	0.0376
		Economic self-reliance rate (C53)	0.0093	0.0227	0.0684
		Farmer satisfaction rate (C54)	0.0093	0.0206	0.0077
		Water quality compliance rate (C55)	0.0007	0.0008	0.0006
		Fund surplus growth rate (C56)	0.0071	0.0727	0.0310

According to Eq 6 and Eq 7, both the positive ideal solution and negative ideal solution of each index are worked out, as shown in Table 5.

**Table 5:** Weights of each indicator and positive and negative ideal solutions.

Overall Layer	System Layer	Indicator Layer	System layer entropy weight (*B*_*i*_)	Indicator layer entropy rights (*C*_*ij*_)	Positive ideal solution (*Y*^*+*^)	Negative ideal solution (*Y*^*-*^)
Performance evaluation of small-scale water resources project management mode (A)	Organization management (B1)	Institutional setting (C11)	0.0702	0.0031	0.0018	0.0013
		Incentive Reward and Punishment Mechanism (C12)		0.0197	0.0136	0.0053
		Supervisory mechanism (C13)		0.0151	0.0101	0.0044
		Democratic Decision-Making Mechanism (C14)		0.0243	0.0171	0.0061
		Archives Management (C15)		0.0079	0.0048	0.0029
	Project Management (B2)	Engineering Property Rights Clarity (C21)	0.3057	0.0015	0.0008	0.0006
		Custody Responsibility Fulfillment Rate (C22)		0.0134	0.0088	0.0041
		Water source engineering integrity (C23)		0.0701	0.0586	0.0062
		Channel uptime (C24)		0.0539	0.0431	0.0072
		Electromechanical equipment uptime (C25)		0.0774	0.0658	0.0055
		Gutter integrity (C26)		0.0895	0.0779	0.0042
	Water Management (B3)	Frequency of water conflicts (C31)	0.1724	0.0526	0.0479	0.0086
		Water cycle reduction rate (C32)		0.0650	0.0521	0.0086
		Irrigation water use efficiency (C33)		0.0130	0.0085	0.0041
		Standardize water registration rate (C34)		0.0315	0.0230	0.0069
		Water Plan Completion Rate (C35)		0.0103	0.0065	0.0034
	Economic Management (B4)	Water Pricing Rationality (C41)	0.1846	0.0136	0.0090	0.0041
		Water charge rate (C42)		0.0270	0.0188	0.0070
		Water price cost ratio (C43)		0.0099	0.0066	0.0036
		Water charges use transparency (C44)		0.0072	0.0045	0.0026
		Fixed asset depreciation rate (C45)		0.1268	0.1104	0.0059
	Sustainable Management (B5)	Water Security Ratio (C51)	0.2671	0.0180	0.0122	0.0051
		Funding Guarantee Rate (C52)		0.0482	0.0376	0.0076
		Economic self-reliance rate (C53)		0.0836	0.0684	0.0093
		Farmer satisfaction rate (C54)		0.0296	0.0206	0.0077
		Water quality compliance rate (C55)		0.0014	0.0008	0.0006
		Fund surplus growth rate (C56)		0.0863	0.0727	0.0071

### Analysis on evaluation results

With the aim of better managing and maintaining the new project, extending the service life of the project, and giving full play to the maximum benefits of the project, Jiaoling County has taken lead in the establishment of a cooperative with the status of a legal person through the village committee and the party branch at village level. They are launched on the basis of giving full play to the active investment and investment of the masses. As an enterprise with the nature of joint-stock cooperative, the small-scale water resources engineering cooperative formulates the management charter based on the shareholding cooperative system. The management layer of this joint-stock cooperative is elected by the shareholders each year. As a consequence, it can not only fully protect the water farmers’ rights to participate and avoid the possibility of some entities’ occupying certain positions for a long time effectively, but also initially have an organizational management mode which is relatively complete. According to the calculation carried out previously, the evaluation of the performance shown by the management mode of small-scale water resources projects in Jiaoling County under the joint-stock system is carried out. The results obtained from specific calculation are shown in Table 6.

**Table 6:** Euclidean spatial distance between each subsystem and the ideal solution under the joint-stock management mode.

Subsystem	Positive ideal solution (*Y*^*+*^)	Negative ideal solution (*Y*^*-*^)	Closeness (*D*_*j*_)	rating
organization and management	0.0045	0.0115	0.7188	Good
project management	0.1146	0.0002	0.0017	Poor
water management	0.0520	0.0139	0.2109	Poor
economic management	0.1050	0.0061	0.0549	Poor
sustainability management	0.0943	0.0017	0.0177	Poor

Obviously, small-scale water resources projects in Gaozhou City, Guangdong Province have been seriously out of control since the 1980s. Having learned the experience from the management of small-scale water resources projects both at home and abroad, Guangdong Provincial Water resources Department has established a management mode dominated by water user association in Zhenjiang Town. It adopts a system of hierarchical responsibility, and the water users association is responsible for the unified management and corresponding ancillary facilities of small-scale water resources projects. The results obtained from specific evaluation are shown in Table 7.

**Table 7:** Euclidean spatial distances between subsystems and ideal solutions in the water-user association model.

Subsystem	Positive ideal solution (*Y*^*+*^)	Negative ideal solution (*Y*^*-*^)	Closeness (*D*_*j*_)	rating
organization and management	0.0020	0.0149	0.8817	Excellent
project management	0.0026	0.1130	0.9775	Excellent
water management	0.0015	0.0607	0.9759	Excellent
economic management	0.0122	0.1046	0.8955	Excellent
sustainability management	0.0483	0.0701	0.5921	medium

Nanhai District adopts a government-dominated management mode for small-scale water resources projects, in which the management agency of small-scale water resources projects is an institution engaged in the process permanently. It is composed of the mayor, the leaders and the representatives. The mayor in charge of the affairs in the town is responsible for the revenue of agriculture in the town. The leaders of relevant departments of business at a higher level and the representatives of each village obtaining income are subject to the management of the government at a higher level. The government-dominated management agency of small-scale water resources projects is composed of the Finance Department, the Cashier, the Project Management Department, the Logistics Support Department and the Comprehensive Support Department. Among them, the Finance Department deals with the business of financial management, documents and archives. Specific evaluation results are listed in Table 8 as follows.

**Table 8:** Euclidean spatial distances between subsystems and ideal solutions in the water-user association model.

Subsystem	Positive ideal solution (*Y*^*+*^)	Negative ideal solution (*Y*^*-*^)	Closeness (*D*_*j*_)	rating
organization and management	0.0149	0.0020	0.1183	Poor
project management	0.0168	0.1026	0.8593	Excellent
water management	0.0479	0.0258	0.3501	medium
economic management	0.0797	0.0277	0.2579	Poor
sustainability management	0.0437	0.0708	0.6183	Good

### Comparative analysis

#### (1) Comparison of management performance at the system level

**Fig 1:**
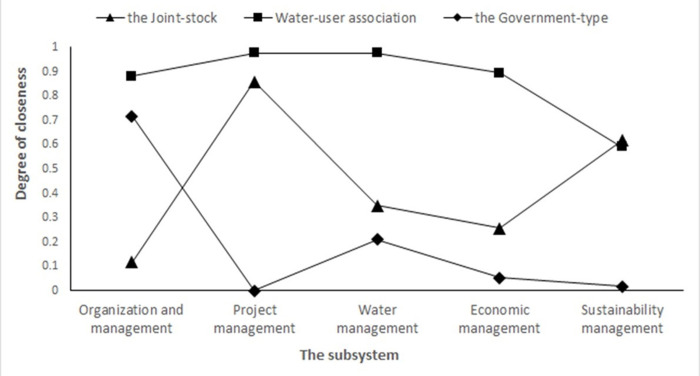
Comparison of closeness degrees under three management modes.

According to the evaluation results from the method of entropy rights, the weights at each system level are sorted out as follows: engineering management subsystem (30.57%) > sustainable management subsystem (26.71%) > economic management subsystem (18.46%) > water management subsystem (17.24%) > organizational management subsystem (7.02%). It is rather clear that the engineering management subsystem and the sustainable management subsystem are two most important subsystems which impose influence on the management performance of small-scale water resources projects. They are followed by the economic management subsystem and the water management subsystem, with the organizational management subsystem ranking at the bottom. As shown in Fig 1, there is a comparison of the evaluation of the three management modes for the three study subjects.

#### (2) Comparison of management performance at overall layer

The results obtained from the evaluation of the improved TOPSIS model show that the closeness of Gaozhou City (0.8817) > the closeness of Jiaoling County (0.7188) > the closeness South China Sea zone (0.1183). Gaozhou City established a relatively complete participatory management mechanism using the Water User Association model. Because of it, the smooth development has provided farmers with a way to get engaged in the management of small-scale water resources projects with an excellent rating. Jiaoling County established a platform of cooperation for various stakeholders through the establishment of cooperatives applying the mode of joint-stock management, and it is easy to see that the evaluation level is favorable. The government-dominated Nanhai District is subject to the uniform management of the engineering management bureau responsible for small-scale water resources established by the government. However, the democratic decision-making mechanisms and the incentive mechanism applied in the process are not yet perfect, and there is a poor level of evaluation accordingly.

Based on the evaluation results obtained from the TOPSIS model, the management performance of small-scale water resources projects under the three management modes is compared. Specific values are listed in the Table 9. In the model of joint-stock system, the value of (*Y*^*+*^), the distance between the overall layer and the positive ideal point, is 0.1892, and the value of (*Y*^*-*^), the distance from the negative ideal point, is 0.0912. Besides, the value of (*D*_1_), which represents closeness, is 0.0919, rated as being bad. In the model of water users association, the value of (*Y*^*+*^) is 0.0500, and the value of (*Y*^*-*^) is 0.1803. Besides, the value of (*D*_2_) is 0.7830, rated as being good. In the government-dominated model, the value of (*Y*^*+*^) is 0.1051, and the value of (*Y*^*-*^) is 0.1303. Moreover, the value of (*D*_3_) is 0.5534, rated as being medium.

**Table 9:** Overall management performance evaluation under three management modes.

Management Mode	Positive Ideal Solution Distance (*Y*^*+*^)	Negative Ideal Solution (*Y*^*-*^)	Closeness (*D*_*j*_)	Sort	Rating
Joint-stock System Mode	0.1892	0.0192	0.0919	3	Poor
Water Users Association Mode	0.0500	0.1803	0.7830	1	Good
Government-led Mode	0.1051	0.1303	0.5534	2	Medium

It is observed that the management performance of the overall layers in the three management modes is ranked as follows: the water users association mode> the government-dominated mode> the joint-stock system mode.

In summary, among the three management modes, the closeness index of the overall layer in the management mode of the water users association is higher than that of the other two regions, which indicates that its overall management performance is superior to that of the other two. The closeness of the four subsystems including organization management, engineering management, water management subsystem, and economic management under the management mode of the water users association is higher than that of the other two as well. In particular, the closeness shown by the two subsystems of water management and economic management is much greater than that of the other two modes. It is shown that the better performance is experienced by the activities of daily water use and economic activities of small-scale water resources projects under the management mode of the water users association.

## Conclusions

Most of the subjects for the management small-scale water resources projects are farmers, while their participation awareness is weak. Therefore, the small-scale water resources projects at the village level are almost out of control. It has turned out to be a universal problem in the new rural construction in our country. On such basis, this paper firstly analyzes the three typical management modes of small-scale water resources projects, followed by the selection of the representative problems in the small-scale water resources project management in general from three management modes. The management mode of small-scale water resources project under shareholding system has imperfect supervision mechanism. With regard to the management mode of water users association, the right of management is separated from the right of maintenance. Under the government-led management mode, property rights are relatively concentrated, with economic and social benefits required to be considered. Then, according to the analysis on three typical modes from special to general, the subsystems set up include the organization management subsystem, project management subsystem, water management subsystem, economic management subsystem, and management performance evaluation index system of sustainable management subsystem. The index system can well apply to a wide range of small-scale water resources project performance evaluation. Then, a performance evaluation model of small-scale water resources project management is constructed. Through the combination of entropy method and TOPSIS model, subjectivity in model construction and weight calculation is reduced. Based on the establishment of the evaluation system and evaluation model, the performance of water resources management is evaluated and analyzed, and the effect of the model in practical application is tested, using the survey data of water resources users, through the combination with the actual situation in the three regions in Guangdong Province. Finally, by comparing the management performance of the three regions at the system level and the overall level, it is concluded that the management model of WUAS is suitable for the development of small-scale water resources projects in Guangdong Province.

From the evaluation results, the management performance evaluation index of small-scale water resources project proposed in this paper can evaluate the general small-scale water resources project objectively based on relevant standards. Compared with the two methods alone, the proposed method combining TOPSIS and entropy weight method has greatly improved its objectivity and accuracy. This method can be applied to not only Guangdong, but also similar small-scale water resources projects around the world. The rational use of evaluation method can allow the small-scale water resources projects in corresponding regions to achieve better management effects. In the future research, we will consider the influence of external engineering factors on management performance, and try to incorporate external factors into the evaluation model to provide a more comprehensive evaluation model.

## Supporting information

S1 Table Additional table (Table 3 Data before standardization). (DOCX)

## References

[1] Kattel GR., Shang W, Wang Z, Langford J. China’s South-to-North Water Diversion Project Empowers Sustainable Water Resources System in the North. SUSTAINABILITY, 2019. 11(13).

[2] Zhao ZH. Guidelines of the Ministry of Water resources on deepening water resources reform. China Water Conservancy, 2014, 03, 1–5. CNKI:SUN:SLZG.0.2014-03-006(in Chinese)

[3] Shuang XZ, Yu GB. Analysis on Construction Management of Small-scale Water resources Projects in the Countryside of China. Proceedings of 2015 2nd International Conference on Education, Management and Information Technology, 2015,153–157.

[4] Du LJ, Xu L, Li YP, Liu CS, Li ZH, Wong JS, et al. China’s Agricultural Irrigation and Water resources Projects: A Policy Synthesis and Discussion of Emerging Issues. Sustainability, 2019,11(24), 7027. doi: 10.3390/su11247027

[5] Feng GZ. Train of Thought Reforming Small-sized Rural Water resources Project. China Rural Water and Hydropower, 2001,08, 1–5. doi: 10.3969/j.issn.1000-1123.2001.08.007(in Chinese)

[6] Mei Y. Research on water resources value of Zichuan District based on grey comprehensive evaluation, in 2ND INTERNATIONAL CONFERENCE ON APPLIED MATHEMATICS, MODELLING, AND INTELLIGENT COMPUTING (CAMMIC 2022), Srivastava H.M. and Chen C.H., Srivastava H.M. and Chen C.H.^Editors. 2022: 2nd International Conference on Applied Mathematics, Modelling, and Intelligent Computing (CAMMIC).

[7] Wang JY, Liu CS, Liu XY, Yao SJ, Yu YH. Research on reform of property right system of small-scale Irrigation and Water resources Project—Theoretical model and practical form. China Water Conservancy, 2015,02, 17–20. CNKI:SUN:SLZG.0.2015-02-013

[8] Xi Z. Design and research of irrigation channel in small-scale farmland water resources project. Engineering Construction and Design, 2022, (20):101–103.

[9] Long ZQ, Xu YM, Zhou YQ, Fan RG. The Management Effect of Small-scale Irrigation and Water resources Facilities from the Perspective of Social Capital: An Analysis of Two Water Users’ Associations in Dangyang, Hubei. China Rural Survey, 2018,02, 16–29.

[10] Gu XY, Chen SJ. Function Optimization of Water Users Association from the Perspective of Rural Land "Division of Three Rights". Journal of Hohai University (Philosophy and Social Sciences), 2020,22(03), 99–104+108. doi: 10.3876/j.issn.16714970.2020.03.013

[11] Greenway J. Water resources management versus the world. AIMS GEOSCIENCES, 2021. 7(4): p. 589–604.

[12] Xie Q, Zhou Y. Sichuan rural small-scale water resources project construction and management system reform analysis. China Rural Water and Hydropower, 2012,08, 9–11. CNKI:SUN:ZNSD.0.2012-08-002(in Chinese)

[13] He SK. Study on the choice of multiple supply model and governance mechanism of rural Water Conservancy. Rural Economy, 2016,04, 91–98. CNKI:SUN:NCJJ.0.2016-04-016

[14] Zhang N, Zuo L, Chen T, Zhang L, Dong HJ. Empirical Analysis Based on the Probit Model on the Smart Water Conservancy Construction and the Participation Willingness of Rural Social Subjects in Arid Areas. Xinjiang Agricultural Sciences, 2020,57(12), 2340–2350. doi: 10.6048/j.issn.1001-4330.2020.12.021

[15] Zhang HY, Jiang WL, Xiang H, He Z, Tao B. The reform of small scale water system mechanism: a case study of yunyang, chongqing. Journal of China Agricultural Resources and Regional Planning, 2017,38(12), 49–55. doi: 10.7621/cjarrp.1005-9121.20171208

[16] Yang YX. The Method and Basis of Dam Location Selection Take an Example of Kariba Dam[C]//. Proceedings of 2017 2nd International Conference on Materials Science, Machinery and Energy Engineering(MSMEE 2017)., 2017:705–708.

[17] Lu CY, Wen F, Yang QY, Chen LL, Zong HM. An Evaluation of Urban Land Use Performance Based on the Improved TOPSIS Method and Diagnosis of Its Obstacle Indicators: A Case Study of Chongqing. Resources Science, 2011,33(03), 535–541. CNKI:SUN:ZRZY.0.2011-03-022

[18] Chen Y, Tang Y. Measuring Uncertainty in the Negation Evidence for Multi-Source Information Fusion. Entropy. 2022; 24(11):1596. (in Chinese) doi: 10.3390/e24111596 36359686 PMC9689623

[19] Sun XB, Guo CL, Cui J. Research on evaluation method of water resources carrying capacity based on improved TOPSIS model. HOUILLE BLANCHE-REVUE INTERNATIONALE DE L EAU, 2020(5): p. 68–74.

[20] Zhang L, Zhang L, Xu Y, Zhou P, Yeh CH. Evaluating urban land use efficiency with interacting criteria: An empirical study of cities in Jiangsu China. LAND USE POLICY, 2020. 90.

[21] Rouyendegh BD, Yildizbasi A, Yilmaz I. Evaluation of retail industry performance ability through integrated intuitionistic fuzzy TOPSIS and data envelopment analysis approach. SOFT COMPUTING, 2020. 24(16): p. 12255–12266.

[22] Miterev MX, Turner JR, Mancini M. The organization design perspective on the project-based organization: a structured review. INTERNATIONAL JOURNAL OF MANAGING PROJECTS IN BUSINESS, 2017. 10(3): p. 527–549.

[23] Anantatmula VS, Rad PF. Role of Organizational Project Management Maturity Factors on Project Success. ENGINEERING MANAGEMENT JOURNAL, 2018. 30(3): p. 165–178.

[24] Su Y, Gao WJ, Guan DJ, Zuo TA. Achieving Urban Water Security: a Review of Water Management Approach from Technology Perspective. WATER RESOURCES MANAGEMENT, 2020. 34(13): p. 4163–4179.

[25] Galli BJ. The Future of Economic Decision Making in Project Management. IEEE TRANSACTIONS ON ENGINEERING MANAGEMENT, 2020. 67(2): p. 396–413.

[26] Chow TC, Zailani S, Rahman MK, Zhang QN, Bhuiyan MA, Patwary AK. Impact of sustainable project management on project plan and project success of the manufacturing firm: Structural model assessment. PLOS ONE, 2021. 16(11). doi: 10.1371/journal.pone.0259819 34818357 PMC8612515

