# Supplementary material for: The performance evaluation of management mode of small water resources projects
Source: PLoS One. 2023 Apr 6;18(4):e0282357. doi: 10.1371/journal.pone.0282357 (PMC10079017; doi:10.1371/journal.pone.0282357)
Supplement: S1 Table — (DOCX) [file pone.0282357.s001.docx]

Additional table(Table 3 Data before standardization)

| Overall Layer | System Layer | Indicator Layer | Jiaoling | Gaozhou | Nanhai |
| --- | --- | --- | --- | --- | --- |
| Performance evaluation of small-scale water resources  project management mode (A) | Organization management (B1) | Institutional setting (C11) | 6.039 | 6.877 | 6.2805 |
|  |  | Incentive Reward and Punishment Mechanism (C12) | 6.5705 | 7.4515 | 5.337 |
|  |  | Supervisory mechanism (C13) | 6.6655 | 7.339 | 5.4745 |
|  |  | Democratic Decision-Making Mechanism (C14) | 7.2705 | 7.5225 | 5.2505 |
|  |  | Archives Management (C15) | 6.013 | 5.8235 | 7.0535 |
|  | Project Management (B2) | Engineering Property Rights Clarity (C21) | 6.761 | 6.181 | 6.366 |
|  |  | Custody Responsibility Fulfillment Rate (C22) | 5.5315 | 7.2925 | 6.4925 |
|  |  | Water source engineering integrity (C23) | 4.4435 | 8.183 | 7.6145 |
|  |  | Channel uptime (C24) | 4.6645 | 8.002 | 7.4245 |
|  |  | Electromechanical equipment uptime (C25) | 4.3565 | 8.2535 | 7.3985 |
|  |  | Gutter integrity (C26) | 4.235 | 8.207 | 8.3535 |
|  | Water Management (B3) | Frequency of water conflicts (C31) | 4.8195 | 8.554 | 6.7135 |
|  |  | Water cycle reduction rate (C32) | 5.7185 | 8.007 | 4.658 |
|  |  | Irrigation water use efficiency (C33) | 6.2345 | 7.2635 | 5.5665 |
|  |  | Standardize water registration rate (C34) | 5.0895 | 7.4465 | 7.654 |
|  |  | WaterPlanCompletion Rate (C35) | 5.6615 | 6.836 | 7.186 |
|  | Economic Management (B4) | Water Pricing Rationality (C41) | 7.2995 | 6.5835 | 5.523 |
|  |  | Water charge rate (C42) | 5.715 | 5.299 | 7.4825 |
|  |  | Water price cost ratio (C43) | 7.336 | 6.2145 | 5.816 |
|  |  | Water charges use transparency (C44) | 5.7785 | 7.0905 | 6.5515 |
|  |  | Fixed asset depreciation rate (C45) | 4.232 | 8.356 | 5.221 |
|  | Sustainable Management (B5) | Water Security Ratio (C51) | 5.425 | 7.3795 | 7.1835 |
|  |  | Funding Guarantee Rate (C52) | 4.787 | 6.276 | 7.9015 |
|  |  | Economic self-reliance rate (C53) | 4.5545 | 5.357 | 8.0915 |
|  |  | Farmer satisfaction rate (C54) | 5.575 | 7.4865 | 5.2945 |
|  |  | Water quality compliance rate (C55) | 6.47 | 6.761 | 6.181 |
|  |  | Fund surplus growth rate (C56) | 6.039 | 6.877 | 6.2805 |
